# Supplementary material for: Mechanisms governing the pioneering and redistribution capabilities of the non-classical pioneer PU.1
Source: Nat Commun. 2020 Jan 21;11:402. doi: 10.1038/s41467-019-13960-2 (PMC6972792; doi:10.1038/s41467-019-13960-2)
Supplement: Supplementary file 7 — Source data [file 41467_2019_13960_MOESM7_ESM.zip › Source_Data/FigureS3/FigureS3J_MotifScanOutput/homerResults/motif18.similar.html]

motif18

## Information for motif18

G
C
T
A
C
T
A
G
A
T
C
G
G
T
C
A
G
T
C
A
C
G
A
T
C
A
T
G
G
A
T
C
  
Reverse Opposite:  

C
T
A
G
G
T
A
C
G
C
T
A
C
A
G
T
C
A
G
T
T
A
G
C
G
A
T
C
C
G
A
T
  

|  |  |
| --- | --- |
| p-value: | 1e-63 |
| log p-value: | -1.463e+02 |
| Information Content per bp: | 1.647 |
| Number of Target Sequences with motif | 3778.0 |
| Percentage of Target Sequences with motif | 28.63% |
| Number of Background Sequences with motif | 7765.4 |
| Percentage of Background Sequences with motif | 21.27% |
| Average Position of motif in Targets | 425.2 +/- 376.7bp |
| Average Position of motif in Background | 319.0 +/- 196.4bp |
| Strand Bias (log2 ratio + to - strand density) | -0.0 |
| Multiplicity (# of sites on avg that occur together) | 1.15 |
| Motif File: | file (matrix) reverse opposite |

### Similar de novo motifs found

|  |  |  |  |  |  |  |  |
| --- | --- | --- | --- | --- | --- | --- | --- |
| Rank | Match Score | Redundant Motif | P-value | log P-value | % of Targets | % of Background | Motif file |
| 1 | 0.762 | C G T A C T A G T C A G T G C A T G C A C A G T T C A G A T G C C G T A C T A G C G A T T A C G C G A T A G C T | 1e-23 | -53.206046 | 6.14% | 3.96% | motif file (matrix) |
